# Choline Compounds of the Frontal Lobe and Temporal Glutamatergic System in Bipolar and Schizophrenia Proton Magnetic Resonance Spectroscopy Study

**DOI:** 10.1155/2018/3654894

**Published:** 2018-11-25

**Authors:** Beata Galińska-Skok, Aleksandra Małus, Beata Konarzewska, Anna Rogowska-Zach, Robert Milewski, Eugeniusz Tarasów, Agata Szulc, Napoleon Waszkiewicz

**Affiliations:** ^1^Department of Psychiatry, Medical University of Białystok, Plac Brodowicza 1, 16-070 Choroszcz, Poland; ^2^Department of Statistics and Medical Informatics, Medical University of Białystok, ul. Szpitalna 37, 15-295 Białystok, Poland; ^3^Department of Radiology, Medical University of Białystok, ul. M. Skłodowskiej-Curie 24A 15-276 Białystok, Poland; ^4^Department of Psychiatry, Faculty of Health Sciences, Medical University of Warsaw, ul. Partyzantów 2/4, 05-802 Pruszków, Poland

## Abstract

**Purpose:**

Modern neuroimaging techniques allow investigating brain structures and substances involved in the pathophysiology of mental disorders, trying to find new markers of these disorders. To better understanding of the pathophysiology and differential diagnosis of schizophrenia and bipolar disorder, this study was conducted to assess the neurochemical alterations in the frontal and temporal lobes in hospitalized patients with schizophrenia and bipolar disorder.

**Methods:**

Twenty-one subjects with schizophrenia (paranoid and differentiated types), 16 subjects with bipolar I disorder (manic, depressive, and mixed episode), and 20 healthy subjects were studied. Magnetic resonance (MR) imaging and proton resonance magnetic spectroscopy (^1^H MRS) were performed on a 1.5 T scanner. Voxels of 8 cm^3^ were positioned in the left frontal and left temporal lobes.

**Results:**

Glx/H_2_O (GABA, glutamine, and glutamate/nonsuppressed water signal) ratios were significantly increased in the left temporal lobe in schizophrenia, but not in bipolar disorder, compared with controls. Cho/H_2_O (choline/nonsuppressed water signal) ratios in the left frontal lobe had a tendency to increase in bipolar disorder and schizophrenia, relative to controls. A lower temporal lobe NAA/H_2_O ratio in mixed than in manic and depressive episode of bipolar patients was also found. No other significant differences were found among three studied groups as regards NAA, Cr, and mI ratios.

**Conclusions:**

Our results partially confirm the role of a glutamatergic system in schizophrenia, however, only in a temporal lobe. We also point to the importance of the choline-containing compounds (marker of cellular density) in the frontal lobe of patients suffering from bipolar disorder and schizophrenia. We also found the deleterious effect of mixed bipolar episode on the integrity and functioning of the temporal lobe. Glutamatergic left temporal spectroscopic changes may potentially help in differential diagnosis of schizophrenia from bipolar disorder.

## 1. Introduction

The etiology of schizophrenia and bipolar disorder has not been yet well recognized. Over 100 years ago, Kraepelin proposed dementia praecox and manic-depressive psychosis as two separate diseases. Nevertheless, these two disorders share several clinical features such as psychotic symptoms, typical onset in young adults and earlier in males, as well as the frequent occurrence of life events prior to the onset or relapse [1]. Fischer and Carpenter suggest that overlapping features such as psychotic symptoms are not decisive in differential diagnosis and in each disorder are rather a syndrome, not a disease entity [2]. Furthermore, the genetic studies show that schizophrenia and bipolar disorder share certain susceptibility genes, e.g., *CACNA1C*, *NT5C2*, and *CCDC68* [3]. The genetic risk for schizophrenia is associated with gray matter volume deficits in the bilateral fronto-striato-thalamic and left lateral temporal regions, whereas the genetic risk for bipolar disorder is specifically associated with gray matter deficits only in the right anterior cingulated gyrus and ventral striatum [4].

Modern neuroimaging techniques allow investigating not only brain structures but also substances involved in pathophysiology of mental disorders. Proton magnetic resonance spectroscopy (^1^H MRS) is a useful tool that detects brain metabolites such as NAA (N-acetylaspartate), a marker for neuronal integrity or neuronal-glial homeostasis; Glx (GABA, glutamine, and glutamate); Cho (choline containing compounds), a measure of cellular density; Cr (creatine plus phosphocreatine), a marker of cellular energy level; and mI (myo-inositol), a marker of brain osmotic balance and glial cells [5, 6]. It is known that not only MRI brain imaging or neurophysiological measures but also the neurochemical changes detected by MRS may be biomarkers of schizophrenia and other neuropsychiatric disorders, e.g., posttraumatic stress disorder, antidepressant treatment response, or binge drinking [7–10].

The meta-analysis of ^1^H MRS studies in schizophrenia revealed lower NAA level in the brain of patients with schizophrenia, primarily in the hippocampus, and in the gray and white matter of the frontal lobe [11]. Also, the systematic review of ^1^H MRS findings in bipolar disorder revealed that NAA levels were lower in euthymic bipolar patients in the frontal lobe and hippocampus and Cho/Cr ratios were higher in the basal ganglia of euthymic patients [12]. Glutamate/glutamine levels were higher in all mood states compared to controls. The combined meta-analyses of neurometabolite alterations in both disorders: schizophrenia and bipolar disorder, revealed that NAA levels were affected in schizophrenia and bipolar disorder [13]. The most consistent findings were decreased NAA levels in the basal ganglia and frontal lobe for schizophrenia, but only in the basal ganglia for bipolar disorder. Cho and Cr levels were not altered in either disorder. There are only few studies which directly compare the spectroscopic measures in both disorders [14–20]. These sparse spectroscopy studies report evidence of decreased neuronal integrity in cortical gray matter in both disorders [21]. In order to get better understanding of (to clarify) the pathophysiology of schizophrenia and bipolar disorder, this study was conducted to assess the neurochemical alterations in the frontal and temporal lobes in hospitalized patients with schizophrenia and bipolar disorder.

## 2. Material and Methods

### 2.1. Subjects

Twenty-one subjects with schizophrenia, 16 subjects with bipolar I disorder, and 20 healthy subjects were studied. Two recruited bipolar patients were unable to tolerate the magnetic resonance scanning, and they were not included to the studied group. All patients were hospitalized in the Department of Psychiatry of the Medical University of Białystok and in other inpatient wards of the Psychiatric Hospital in Choroszcz. The diagnosis of schizophrenia and bipolar disorder was made according to the ICD-10 and DSM-IV criteria. The schizophrenic subjects were paranoid type (*n* = 17), and undifferentiated type (*n* = 4). In this group, the clinical symptoms were assessed by the battery of psychiatric measures: Positive and Negative Syndrome Scale (PANSS) [22], Calgary Depression Scale for Schizophrenia (CDSS) [23], and Clinical Global Impression (CGI) [24]. The bipolar patients were in manic episode (*n* = 6), depressive episode (*n* = 5), and mixed episode (*n* = 5). In these patients, the mental state was assessed by the clinical scales: the Montgomery-Asberg Depression Rating Scale (MADRS) [25] and the Young Mania Rating Scale (YMRS) [26]. There were no significant age differences between groups (Table 1). Disease duration was similar in the schizophrenia and bipolar groups. Schizophrenic patients were receiving neuroleptics, and bipolar patients were treated with mood stabilizers, neuroleptics, and antidepressants due to their clinical condition. The study exclusion criteria were as follows: central nervous system organic damage confirmed in a routine neurological and MR examinations, active alcohol and other psychoactive substance dependence, and contraindications to conduct MR examinations. The subjects signed a written approval to participate in the study, in accordance with the protocol approved by the Local Bioethical Committee.

### 2.2. MRI and ^1^H MRS

**Figure 1:**
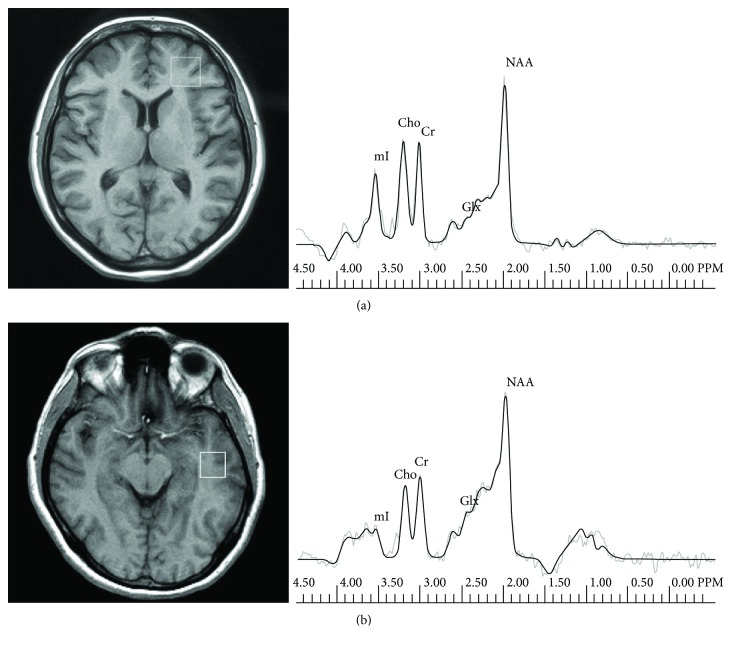
Voxel placement in the frontal lobe with a corresponding representative proton spectrum of a patient with bipolar disorder (a) and voxel placement in the temporal lobe with a corresponding representative proton spectrum of a patient with schizophrenia (b).

MR imaging and MR spectroscopy examinations were performed at the Department of Radiology, Medical University of Białystok, on a 1.5 T scanner (Picker Eclipse, Picker International Inc., Highlands Heights, OH, USA) by means of a standard circularly polarized head coil. T1-weighted FAST scans and conventional FSE T2-weighted series were obtained [27]. ^1^H MR spectroscopy examinations were carried out by means of single voxel PRESS (point-resolved single voxel localized spectroscopy) with the following parameters: TR = 1500 ms, TE = 35 ms (TE1 = ~17 ms), and nex = 192 and 3906 KHz bandwidth. Voxels of 2 × 2 × 2 cm^3^ were positioned in the regions of the left frontal lobe and left temporal lobe. A trained investigator located voxels by eye by means of T1-weighted sections in sagittal, coronal, and axial planes, and the inclusion of CSF was minimized. The left frontal lobe voxel was localized in the region which included the superior and middle frontal gyri and the above anterior horns of the lateral ventricles and is comprised most of all white matter and cortex. The left temporal lobe voxel was localized in the region which included the middle and inferior temporal gyri (Figure 1). Next, the signal over the voxel was shimmed to within a linewidth of 3 to 7 Hz and the transmitter pulse power was optimized by automated procedures. The MOIST (Multiply Optimized Insensitive Suppression Train) method was applied in order to suppress the signal from water [28]. The software package via 2.0C provided by Picker was used to analyse spectroscopic data. The ^1^H MRS data were zero-filled to 8192 points, and residual water resonances were removed using time-domain high-pass filtering. Exponential to Gaussian transformation was applied as a time-domain apodizing Gaussian filter. Next, data were Fourier transformed and phase corrected. After the application of a Legendre polynomial function to approximate the baseline, an automated curve fitting was performed using an iterative, nonlinear least-square fitting procedure by means of the Levenberg-Marquardt algorithm. Line shapes of the simulated peaks used in the fitting process were fixed with 85% Gaussian and 15% Lorentzian fractions. The following metabolites were assessed: NAA (N-acetylaspartate) at 2.01 ppm, Glx (GABA, glutamine, and glutamate) in the area from 2.11 ppm to 2.45 ppm, Cho (choline-containing compounds) at 3.22 ppm, Cr (creatine plus phosphocreatine) at 3.03 ppm, and mI (myo-inositol) at 3.56 ppm (Figure 1). Then metabolite to creatine ratios were analysed as well; the ratio of metabolites to nonsuppressed water signal was calculated according to the following formula: metabolite area × 1000/nonsuppressed water area.

### 2.3. Statistical Analysis

Due to the small size of each group, we performed nonparametric tests. Demographic and clinical data were compared across groups using the Mann–Whitney *U* test, chi-square test, and Kruskal-Wallis test. When the group factor in the Kruskal-Wallis test was significant, post hoc paired comparisons were made. Also, the relationship between metabolic data and age with Spearman's correlation was analysed. Statistical analysis was performed using Statistica 10.0 PL, StatSoft Polska Ltd. The level of significance *P* was assumed to be below 0.05.

## 3. Results

Except for a higher NAA/H_2_O ratio of the temporal lobe in manic than in mixed (*p* = 0.007) and in the depressive than in mixed (*p* = 0.031) episode of bipolar disorder, we found no other differences in any of the studied metabolites between different episodes of bipolar disorder. We also found no differences in any of the studied metabolites between paranoid and undifferentiated types of schizophrenia. Therefore, we included manic, depressive, and mixed episode as a one bipolar group and a paranoid and undifferentiated schizophrenia as a one schizophrenia group to the main statistical analyses.

The left frontal and temporal lobe MRS metabolite ratios are presented in Table 2. Results of the Kruskal-Wallis test showed a significant overall group effect for the Glx/H_2_O ratio in the left temporal lobe (*p* = 0.02). The post hoc tests revealed that Glx/H_2_O ratios were significantly increased only in schizophrenic patients as compared to controls (*p* = 0.02).

Additionally, results of the Kruskal-Wallis test showed a significant overall group effect for the Cho/H_2_O ratio in the left frontal lobe (*p* = 0.04). The post hoc tests revealed only a trend toward a significant increase in the frontal Cho/H_2_O ratio of patients with bipolar disorder and schizophrenia relative to controls (*p* = 0.07 and *p* = 0.08, respectively), and no significant differences between the bipolar and schizophrenia groups. No significant differences were found among the three studied groups as regards NAA, Cr, and mI ratios.

The age of bipolar patients was inversely significantly correlated with the left frontal NAA/H_2_O ratio (*R* Spearman = −0.636; *p* = 0.01). The age of controls significantly correlated with the left frontal Glx/H_2_O ratio (*R* = 0.486; *p* = 0.04).

## 4. Discussion

In our study, we found an increase in Glx/H_2_O ratios in the left temporal lobe in schizophrenic patients relative to controls. Glutamate is the most abundant amino acid in the brain and in the spectral peaks is often grouped together with glutamine and GABA because of their overlaps [5]. Öngür et al. [16] examined the glutamine/glutamate ratio in acute mania, schizophrenia, and controls. In this study, the glutamine/glutamate ratio was significantly higher in the anterior cingulate cortex and parieto-occipital cortex in bipolar disorder, but also, there was a trend toward the significance in schizophrenia, compared with healthy controls. In another study, Atagün et al. [19] observed that glutamate levels were lower in left Heschl's gyrus and the planum temporale for schizophrenia at trend levels compared to healthy participants and glutamate and NAA levels were lower in euthymic bipolar patients when compared to controls. In the review regarding glutamate-related abnormalities in mood disorders [29], a highly consistent pattern of Glx reductions in major depressive disorder and elevations in bipolar disorder is pointed out. Also, depressive and manic episodes may be characterized by modulation of the glutamine/glutamate ratio in opposite directions. Our study group of bipolar patients consisted of manic, mixed, and depressive patients, and no differences in glutamatergic metabolites were found between these groups and between the bipolar group and controls. However, we found the lowest NAA/H_2_O ratio (marker of neuronal integrity) in the temporal lobe in the mixed episode of bipolar disorder, when compared to manic and depressive episodes. It suggests that of bipolar episodes, mostly, the mixed episode may potentially disturb the integrity of functioning of the temporal lobe. In our previous study, we found increased Glx level in the left temporal lobe in first-episode schizophrenic patients as compared to healthy controls [30]. Marsman et al. [31] in review revealed that frontal region glutamate is lower and glutamine is higher in patients with schizophrenia compared to healthy individuals. Our results therefore point to the importance of the glutamatergic system in schizophrenia; however, we observed its importance only in the temporal lobe.

We also observed a trend toward the increase in the frontal Cho/H_2_O ratios in patients with bipolar disorder and schizophrenia, compared to controls. The results of previous studies concerning Cho level among schizophrenia, bipolar, and controls are inconsistent. There were not found differences among these groups in the Cho/Cr ratio in the prefrontal cortex [14], in the anterior cingulate cortex, and he parieto-occipital cortex [16]. On the other hand, Sarramea Crespo et al. [15] observed in schizophrenia patients higher Cho/Cre ratios in the anterior cingulum as compared with controls and bipolar patients. Cho levels in the dorsolateral prefrontal cortex were noted to be lower in patients with schizoaffective and bipolar disorders compared to the control group, and there was no significant difference between the schizophrenic patients and the control group [18]. The findings from our study are consistent with previous studies in bipolar disorder in higher choline levels, reported in the hippocampus [32, 33], in the orbitofrontal cortex [33], and in the anterior cingulate [34]. An increase in the choline-containing compound signal reflects an increase in membrane turnover, myelination, or inflammation [5].

Several limitations have to be taken into account while interpreting the results of our study. Firstly, the use of antipsychotic medication can influence the brain metabolism as measured by the ^1^H MRS [35]. Antipsychotic treatment may increase NAA levels during the -time observation; however, this effect may disappear in longer observation. Also, glutamate measures are decreasing along with the duration of the disease [35]. It was also observed that the treatment of mania with olanzapine may increase choline level [36] or antidepressant use in bipolar disorder is correlated with lower choline levels [34]. The lithium treatment may also influence lower Glx levels and increased myo-inositol in grey matter [37]. Secondly, we did not make segmentation within the volume of interest. This procedure was not available in our study, and thus, we cannot exclude the possibility of small regional variations in metabolite concentration [38]. Nevertheless, the use of nonsuppressed water signal as an internal standard enhances the meaning of our results since reduction in Cr levels is observed in schizophrenia, but not in bipolar disorder, as compared to controls [16]. Then the age is an important factor influencing metabolite ratios, mostly NAA [39]. Also, in our study, we observed the effect of the age on frontal NAA ratios but only on bipolar patients (negative correlation). The meta-analysis of 1H MRS studies found that glutamate and glutamine levels in the frontal region decrease at a faster rate with the age of patients with schizophrenia as compared with healthy controls [31]. In our study, frontal Glx ratios positively correlated with age only in controls, but not in patients.

## 5. Conclusion

Our results point to the role of the temporal glutamatergic system in schizophrenia and choline-containing compounds in both bipolar disorder and schizophrenia. The lowest NAA/H_2_O ratio in the mixed episode of bipolar disorder suggests the deleterious effect of mixed episode on the integrity and functioning of the temporal lobe. Especially, glutamatergic spectroscopic changes may potentially help in the differential diagnosis of bipolar disorder from schizophrenia. The observed alterations in neurometabolites may be involved in the pathogenesis of bipolar disorder and schizophrenia.

## Figures and Tables

**Table 1:** Clinical data for patients with schizophrenia and bipolar disorder, and controls (means ± SD).

	Schizophrenia *N* = 21	Bipolar disorder *N* = 16	Controls *N* = 20	*P* value
Age (years)	37.76 ± 8.04	43.69 ± 11.00	36.95 ± 7.41	0.11^a^
Females/males	11/10	11/5	10/10	0.48^b^
Disease duration (years)	13.33 ± 8.27	8.78 ± 8.37	—	NS^c^
PANSS	86.28 ± 11.02	—	—	
CDSS	4.52 ± 4.52	—	—	
CGI	4.52 ± 0.68	—	—	
MADRS		14.00 ± 12.95		
YMRS		11.06 ± 8.49		

^a^Kruskal-Wallis test. ^b^Chi-square test. ^c^Mann–Whitney *U* test. NS: nonsignificant; PANSS: Positive and Negative Syndrome Scale; CDSS: Calgary Depression Scale for Schizophrenia; CGI: Clinical Global Impression; MADRS: Montgomery-Asberg Depression Rating Scale; YMRS: Young Mania Rating Scale.

**Table 2:** Metabolite ratios in the frontal and temporal lobes for patients with schizophrenia and bipolar disorder and controls (means ± SD).

Metabolite ratios	Patients with schizophrenia *N* = 21	Patients with bipolar disorder *N* = 16	Controls *N* = 20	*P* value
Left frontal lobe				
NAA/Cr	1.75 ± 0.22	1.75 ± 0.32	1.74 ± 0.21	NS
Glx/Cr	1.98 ± 0.29	1.93 ± 0.56	2.17 ± 0.39	NS
Cho/Cr	0.99 ± 0.17	0.99 ± 0.12	0.90 ± 0.17	NS
mI/Cr	0.76 ± 0.26	0.78 ± 0.19	0.71 ± 0.25	NS
NAA/H_2_O	0.47 ± 0.08	0.47 ± 0.08	0.46 ± 0.06	NS
Glx/H_2_O	0.54 ± 0.08	0.50 ± 0.12	0.56 ± 0.10	NS
Cho/H_2_O	0.26 ± 0.04	0.27 ± 0.05	0.23 ± 0.04	0.04
Cr/H_2_O	0.27 ± 0.02	0.26 ± 0.05	0.26 ± 0.03	NS
mI/H_2_O	0.20 ± 0.07	0.21 ± 0.05	0.18 ± 0.06	NS
Left temporal lobe				
NAA/Cr	1.83 ± 0.37	1.73 ± 0.36	1.78 ± 0.35	NS
Glx/Cr	2.24 ± 0.44	2.18 ± 0.58	2.24 ± 0.66	NS
Cho/Cr	0.98 ± 0.17	0.97 ± 0.20	1.00 ± 0.20	NS
mI/Cr	0.73 ± 0.22	0.73 ± 0.27	0.72 ± 0.24	NS
NAA/H_2_O	0.45 ± 0.11	0.39 ± 0.07	0.40 ± 0.08	NS
Glx/H_2_O	0.62 ± 0.07	0.53 ± 0.14	0.54 ± 0.15	0.02
Cho/H_2_O	0.25 ± 0.06	0.23 ± 0.07	0.23 ± 0.06	NS
Cr/H_2_O	0.25 ± 0.06	0.24 ± 0.04	0.23 ± 0.05	NS
mI/H_2_O	0.18 ± 0.06	0.17 ± 0.06	0.17 ± 0.07	NS

Kruskal-Wallis test. NS: nonsignificant; NAA: N-acetylaspartate; Glx: GABA, glutamine, and glutamate; Cho: choline-containing compounds; Cr: creatine plus phosphocreatine; mI: myo-inositol.

## Data Availability

The data used to support the findings of this study are available from the corresponding author upon request.
